# Report on the Archetype and Homologies of the Vertebrate Skeleton

**Published:** 1847-10-01

**Authors:** 


Report on the Archetype and Homologies of the Vertebrate Skeleton. By Professor *Owen*, F.R.S., &c. (From the Report of the British Association for the Advancement of Science for 1846). 8vo. pp. 171. London: R. and J. Taylor, 1847.

Having, in the review of the distinguished author's Lectures on the Comparative Anatomy and Physiology of Vertebrate Animals, in our last number, made the reader acquainted with the more prominent features of his theory of the cranial vertebræ, it is unnecessary to do more, upon the present occasion, than merely recommend this Report to the studious perusal of every one who feels an interest in the philosophy of osteological science. Even if space permitted, a lengthened notice of its contents would not be exactly suited to the pages of a Journal whose main object is the advancement of practical medicine. To show, however, that such enquiries, notwithstanding their subtle and somewhat abstruse nature, are not wholly devoid of interest to him who is engaged in the exercise of the healing art, we gladly find a place for the following observations. Most of the terms used in it will be found explained in our last number.

"The abnormal conditions of the human skull give further illustration of the truth of the general homologies of the cranial bones, and reciprocally receive light from such determinations. In the case of idiots from defective growth or development of the brain, where the cavity of the cranium is reduced to half or less than half its normal capacity, as *e. g.* in the skull described and figured in my 'Memoir on the Osteology of the Chimpanzee,' it might have been expected from the anthropotomical ideas of the cranial bones,—according to which no one bone is deemed either more or less important than another in its essential nature, and where the squamosal is as little regarded in the light of a superadded or intercalary piece as the alisphenoid,—that all would be reduced in the same proportion in forming the parietes of the contracted brain-chamber. But this is by no means the case. In the instance above-cited the basioccipital and basisphenoid have been developed to their usual size, and the distance from the posterior boundary of the bony palate to the anterior border of the foramen magnum is as great as in any normal skull. The exoccipitals (condyloid portions of the occiput), the alisphenoids and the orbitosphenoids retain in like manner their full dimensions. The distance between the frontal and temporal bones is as great as in the average of fully developed Caucasian skulls, and is greater than in most of those from the Melanian race, in which the direct junction of the frontal with the temporal, as in the chimpanzee, is by no means rare. The contraction of the capacity of the brain-chamber is due chiefly to arrested development of the frontals, parietals, supraoccipital and squamosals. By the reduction of the supraoccipital and the retention of the centrums of the cranial vertebræ of their normal proportions, the foramen magnum becomes situated nearer the back part of the basis cranii than in the normal skull.

"In a still smaller cranium of a female idiot, who reached the age of twenty-one years, which is preserved with the male idiot's skull above-mentioned in the anatomical museum of St. Bartholomew's Hospital, the contrast between the normal proportions of the basioccipital, basisphenoid, exoccipitals, alisphenoids and orbitosphenoids, on the one hand, and the reduced dimensions of the supraoccipital, parietals, frontals and squamosals on the other, is still more striking and significant of the true nature of those bones. The normal growth of the centrums, indeed, might be explained by the concomitant nearly normal size of the medulla oblongata, base of third ventricle and optic chiasma, in the brain of the same idiot: but it is not so obvious from the condition of the brain itself why the alisphenoid should not have shrunk in the same proportion as the parietals, frontals and squamosals. To the homologist, however, the recognised difference of subjectivity to modification presented by the neurapophyses, spines and diverging appendages of the typical segments, renders very intelligible the partial seats of arrested growth in the bones of these idiots' crania.

"In reference to disease, also, one sees not why the alisphenoid should have a minor attraction for the morbid products deposited, or be less subject to the destructive actions excited during syphilitic or mercurial disease, than the parietals, or the orbitosphenoids than the frontals, or the exoccipitals than the supraoccipital; yet it needs but to examine any series of such morbid skulls in our museums of pathology to be convinced that the variable and peripheral elements of the neural arches, viz. their expanded spines, are almost exclusively so affected: the frontal and parietal being the most common seats of the disease: the supraoccipital a less frequent one, concomitantly with its minor deviation from the typical standard of the element. I have yet seen no example in which either a cranio-vertebral centrum or neurapophysis was so affected; but the nasal bones are notoriously attacked."

